# Pancarpal dissociation, a very rare type of injury: A case report

**DOI:** 10.1097/MD.0000000000029479

**Published:** 2022-06-17

**Authors:** Ho Youn Park, Yoo Joon Sur, Dohyung Lim, Kwansoo Lee, Il-Jung Park

**Affiliations:** a Department of Orthopaedic Surgery, Uijeongbu St. Mary's Hospital, College of Medicine, The Catholic University of Korea, Seoul, Republic of Korea; b Department of Mechanical Engineering, Sejong University, Seoul, Republic of Korea; c Department of Orthopaedic Surgery, Bucheon St. Mary's Hospital, College of Medicine, The Catholic University of Korea, Seoul, Republic of Korea.

**Keywords:** capitate fracture-dislocation, hamate dislocation, lunotriquetral dissociation, pan-carpal dissociation, scapholunate dissociation

## Abstract

**Rationale:**

Pan-carpal dissociation is very rare injury and there is little information as to diagnosis, treatment, and prognosis of this injury.

**Patient concerns:**

A 35-year-man presented to our hospital with severe pain and swelling of the left wrist and forearm after slipping and falling while riding a motorcycle.

**Diagnosis:**

The wrist simple radiographs demonstrated unrecognizable severe fracture-dislocation of the carpal bones concomitant with fractures of the radioulnar shaft. Three-dimensional computed tomography revealed a capitate fracture-dislocation, as well as hamate dislocation, lunotriquetral (LT), and scapholunate (SL) dissociation. These findings suggested pan-carpal dissociation.

**Interventions:**

To prevent compartment syndrome, fasciotomy, carpal tunnel release, and open reduction and plate fixation for both bone fracture were performed first. Then, for pan-carpal dissociation, the capitate, carpometacarpal joint (CMCJ), and hamate were reduced and fixed first. Then, the SL, LT, and other intercarpal ligaments were repaired. Finally, additional trans-carpal pins to reinforce the ligament repair and 2.0 mm plate to buttress the third CMCJ were fixed. The patient was instructed to begin gentle range of motion exercises of the wrist with pins from four weeks after surgery and all pins were removed at six weeks postoperatively.

**Outcomes:**

12 months after the operation, the patient exhibited almost full range of motion with mild pain with VAS (Visual analogue scale) 1–2 at rest and VAS 3–4 with effort. Quick DASH (the disabilities of the arm, shoulder and hand) score was 25 and modified Mayo score was 70. The radiographs demonstrated union of the radioulnar shaft, and the carpal bone alignment was successfully maintained

**Lessons:**

Pan-carpal dissociation can be diagnosed in patients with capitate fracture-dislocation, hamate dislocation, LT, and SL dissociation. This pattern of injury is very rare and the authors recommend reduction and fixation of the distal carpal row, followed by the proximal row to facilitate an easy approach to the distal carpal row. Although it is very severe injury, rigid anatomical fixation and an early rehabilitation can lead to favorable functional outcomes.

## 1 Introduction

Carpal dislocation and fracture-dislocation are usually caused by high-energy trauma.^[1]^ Perilunate injury according to a classification described by Mayfield is well-known injury pattern, but there are many variations that are not clearly categorized.^[2–4]^ Among these, pancarpal dissociation is even rarer and has not been clearly defined. Only one case report has been published, but this case was more consistent with capitate fracture-dislocation than pancarpal dissociation.^[5]^

Diagnosis of this type of injury may be difficult and treatment can be challenging. Pancarpal dissocation may be regarded as most severe form of perilunate injury, however, it is different because it involves more complex injuries to the distal carpal row. To the authors’ knowledge, there has been no report of a case with pancarpal dissociation. The authors experienced a patient with “pancarpal dissociation” and would like to share this injury with a review of the relevant literature.

## 2 Case presentation

**Figure 1:**
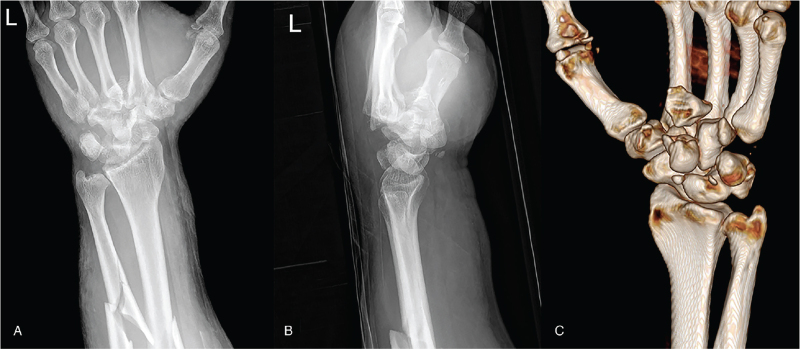
The radiographs demonstrated severe fracture-dislocation of the carpal bones and fractures of the radioulnar shaft (A, B). Three-dimensional image suggest pancarpal dissociation (C).

A 35-year-man presented to our hospital with severe pain and swelling of the left wrist and forearm after slipping and falling while riding a motorcycle. The wrist posteroanterior and lateral view demonstrated unrecognizable severe fracture-dislocation of the carpal bones, and fractures of the radioulnar shaft. Three-dimensional computed tomography (3DCT) revealed a capitate fracture-dislocation, as well as hamate dislocation, lunotriquetral (LT), and scapholunate (SL) dissociation (Fig. 1). These findings suggested pancarpal dissociation.

**Figure 2:**
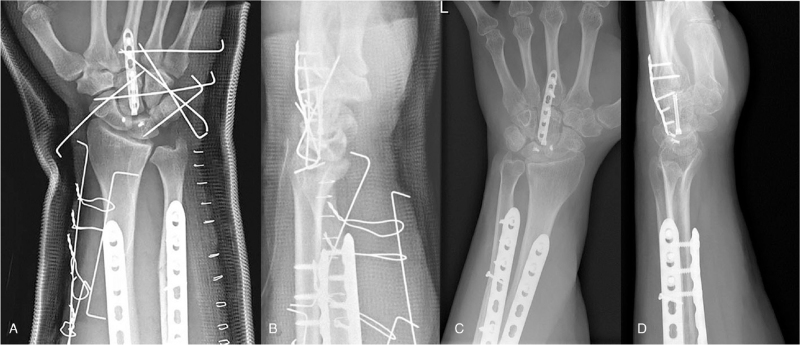
The capitate, CMCJ, and hamate were reduced and fixed. The SL and LT ligaments were repaired using two anchor sutures (black revere triangles), and other intercarpal ligaments were repaired. Transcarpal pins were inserted and a 2.0 small plate was fixed to buttress the CMCJ. Immediate (A, B) and 12 months (C, D) postoperative radiographs demonstrated well reduced carpal alignment. CMCJ = carpometacarpal joint.

Surgery was performed on the day of the injury to prevent compartment syndrome. Fasciotomy, open reduction, and internal fixation using 3.5 mm LCP plates (Synthes Inc., West Chester, PA) were performed on his forearm fractures. Since the forearm swelling was severe, wiring sutures were applied to the volar-side of the forearm for gradual closure. To address the pancarpal dissociation, a longitudinal incision was made at the dorsum of the wrist. First, the capitate was reduced and fixed with a 1.1 mm Kirschner wire (K-wire). Second, the third to fifth carpometacarpal joint (CMCJ) and hamate were reduced and fixed with the K-wires. Then, a 3.0 Headless Compression Screw (Synthes Inc.) was inserted to rigidly fixate the capitate. Third, the SL and LT ligaments were repaired using two mini Mitek devices, and the other intercarpal ligaments were repaired with 2-0 Ethibond sutures. Finally, additional transcarpal pins were inserted to reinforce the ligament repair, and the 2.0 mm LCP (Synthes Inc.) was fixed to buttress the third CMCJ (Fig. 2). Five days after the initial operation, a full-thickness skin graft was performed to cover the skin defect of the forearm.

Postoperatively, an above-elbow splint was applied with the forearm in neutral position. Two weeks postoperatively, the splint was changed to a short-arm splint. The patient was instructed to begin gentle range of motion exercises of the wrist with pins from four weeks after surgery and all pins were removed at six weeks postoperatively.

Twelve months after the operation, the radiographs demonstrated union of the radioulnar shaft, and the carpal bone alignment was successfully maintained (Fig. 2). There was no complication such as infection or metal failure. The patient exhibited almost full range of motion of the wrist, forearm, and hand. The patient had mild pain (VAS 1-2) at rest and (VAS 3-4) with effort. Electromyography and nerve conduction examination revealed mild carpal tunnel syndrome, ulnar and radial neuropathy (partial axonotmesis) in the injured area. The symptoms were not debilitating, and the patient was under observation as he recovered from the neuropathy.

## 3 Discussion

Acute carpal instability has numerous variations and pancarpal dissociation may be the most severe form of injury. Because of the rarity of this injury, however, pancarpal dissociation has not been clearly defined so far and there is no standard treatment. The patient presented here showed combination of perilunate dislocation, capitate fracture-dislocation, and hamate dislocation and the authors believe that this pattern can be regarded as pancarpal dissociation.

Among the carpal bones, the capitate has inherent stability because of its cuboid shape, central location, and the surrounding ligaments.^[6]^ A significant amount of force is required to create additional fractures and dislocation of the capitate that accompany perilunate injury or other complex carpal abnormalities. A typical perilunate injury associated with capitate injury is scaphocapitate syndrome. This injury is defined as combination of a scaphoid fracture and a capiate fracture which was reported as a unique form of carpal bone injury.^[7]^ Our case is similar to scaphocapitate syndrome, but it also had hamate dislocation, which were different from scaphocapitate syndrome.

Hamate dislocation is also very rare injury and has been classified under axial carpal instability.^[8,9]^ Axial disruptions are mostly reported to be caused by industrial crushing injuries such as punch or rolling presses machines.^[10]^ Our case had hamate dislocation but axial injury pattern was not observed. To the authors’ knowledge, this is the first case of hamate dislocation associated with perilunate injury and capitate fracture-dislocation. Although the mechanism of injury is unclear, it is believed that a significant amount of force after a fall while riding a high-speed motorcycle caused hyperextension and crushing of the palm and wrist, causing pancarpal dissociation.

Although surgical treatment is usually indicated, the treatment strategy for pancarpal dissociation has not been standardized, and it is challenging. The authors recommend performing the surgery in the following order. First, the displaced capitate is reduced and fixed to prevent avascular necrosis. Next, the CMCJ, hamate are reduced and fixed. Then, proximal row is treated sequentially. Ting et al.^[5]^ discussed wrist fusion as a treatment for pancarpal dissociation, but in this case, anatomical reduction was performed. One year after surgery, the patient had near-full range of motion, and radiographs showed adequate carpal bone alignment.

In this brief communication, the authors shared a rare case of pancarpal dissociation demonstrated surgical methods. Pancarpal dissociation can be defined as combination of perilunate dislocation, capitate fracture-dislocation, and hamate dislocation. The authors recommend reduction and fixation of the distal carpal row, followed by the proximal row. This sequence facilitates an easy approach to the distal carpal row. Although it is very severe injury, an early rehabilitation can lead to favorable functional outcomes.

## Author contributions

**Conceptualization:** Ho Youn Park, Il-Jung Park.

**Data curation:** Ho Youn Park.

**Supervision:** Dohyung Lim.

**Validation:** Yoo Joon Sur.

**Visualization:** Ho Youn Park.

**Writing – original draft:** Kwanwoo Lee.

**Writing – review & editing:** Il-Jung Park, Ho Youn Park.
